# Persistent opioid use in cataract surgery pain management and the role of nonopioid alternatives

**DOI:** 10.1097/j.jcrs.0000000000000860

**Published:** 2021-11-12

**Authors:** Richard S. Davidson, Kendall Donaldson, Maggie Jeffries, Sumitra Khandelwal, Michael Raizman, Yasaira Rodriguez Torres, Terry Kim

**Affiliations:** From the University of Colorado Eye Center, Denver, Colorado (Davidson); the Bascom Palmer Eye Institute, Plantation, Florida (Donaldson); the Houston Eye Associates, Houston, Texas (Jeffries); the Eye Center of Texas, Houston, Texas (Jeffries); the Baylor College of Medicine, Cullen Eye Institute, Houston, Texas (Khandelwal); the Tufts University School of Medicine, New England Eye Center, Boston, Massachusetts (Raizman); the Kresge Eye Institute, Detroit, Michigan (Rodriguez Torres); the Elmquist Eye Group, Fort Myers, Florida (Rodriguez Torres); the Duke Eye Center, Duke University, Durham, North Carolina (Kim).

## Abstract

Cataracts are a leading cause of preventable blindness globally. Although care varies between developing and industrialized countries, surgery is the single effective approach to treating cataracts. From the earliest documented primitive cataract removals to today's advanced techniques, cataract surgery has evolved dramatically. As surgical techniques have developed, so have approaches to surgical pain management. With current cataract surgical procedures and advanced technology, anesthesia and intraoperative pain management have shifted to topical/intracameral anesthetics, with or without low-dose systemic analgesia and anxiolysis. Despite this, pain and discomfort persist in some patients and are underappreciated in modern cataract surgery. Although pain management has progressed, opioids remain a mainstay intraoperatively and, to a lesser extent, postoperatively. This article discusses the evolution of pain management in cataract surgery, particularly the use of opioids and the associated risks as well as how ophthalmology can have a positive impact on the opioid crisis.

## Abstract 2

Cataract surgery pain management in light of the opioid crisis and how ophthalmologists can help make a positive impact with the use of non-opioid options is discussed.

The United States is in the midst of an opioid epidemic. The use of opioids is increasing, and those most at risk are individuals over age 50 years.^1–3^ This is also the population in which cataract surgery, the most common outpatient surgical procedure worldwide, is most frequently performed.^4–9^ Opioids, which carry a high risk for addiction and overdose, are used perioperatively in cataract surgery anesthesia protocols and postoperatively for pain management in some cases. When considered collectively, the use of opioids in this cataract surgery population creates a perfect storm for potential opioid use disorder (OUD). The literature lacks a comprehensive review of opioids and their relationship to cataract surgery. This article discusses the evolution of cataract surgery and associated pain management, particularly the role and use of opioids in cataract surgery, the associated risks of opioid use in this vulnerable population, and how ophthalmologists and optometrists can help combat the opioid crisis by using nonopioid alternatives to manage pain in their cataract surgery patients.

## EVOLUTION OF CATARACT SURGERY TECHNIQUES

**Figure 1.**
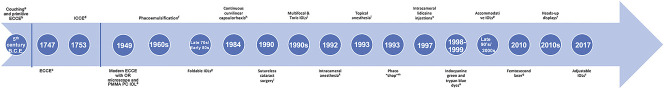
Evolution of cataract surgery techniques. References: ^a^^4^–^9^; ^b^^5^,^6^; ^c^^5^,^6^,^8^,^11^,^12^; ^d^^5^,^6^,^11^–^13^; ^e^^5^,^6^,^8^,^9^,^11^,^14^; ^f^^6^,^11^,^15^,^16^; ^g^^6^,^11^,^14^; ^h^^11^,^17^; ^i^^18^; ^j^^5^; ^k^^19^; ^l^^6^; ^m^^11^; ^n^^8^; ^o^^11^; ^p^^5^,^6^,^14^; ^q^^5^,^6^; ^r^^20^; ^s^^21^. ECCE = extracapsular cataract extraction; ICCE = intracapsular cataract extraction; OR = operating room; PC = posterior chamber

From couching in the fifth century BCE, a procedure in which the lens is dislocated into the vitreous via a needle, to today's phacoemulsification procedures, cataract surgery has become the most common surgery worldwide.^4–9^ Phacoemulsification is now performed in 96% to 97% of all cataract surgeries in the United States.^8,10,11^ Further advances such as femtosecond lasers, premium intraocular lens, pupil expansion devices, intraoperative dyes, and options for topical anesthesia have made today's cataract surgery elegant and safe with a very high success rate (Figure 1).^4–9,11–21^

## EVOLUTION OF PAIN MANAGEMENT/ANESTHESIA

**Figure 2.**
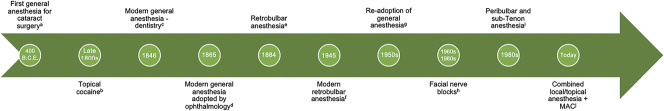
Advances in cataract surgery pain management. References: ^a^^22^; ^b^^12^,^22^,^23^; ^c^^12^; ^d^^12^; ^e^^12^,^23^,^27^; ^f^^12^,^23^,^24^,^28^; ^g^^12^,^25^; ^h^^12^,^23^; ^i^^12^,^23^,^24^; ^j^^12^,^26^. MAC = monitored anesthesia care

Cataract surgery pain management has also advanced. Since the ancient Egyptians and Assyrians used carotid compression to render patients unconscious for cataract surgery, anesthesia and pain management have included a range of general, regional, and topical approaches.^12,22–25^ With newer surgical techniques, instrumentation, and technology, most cataract surgery is performed using a combination of local and topical anesthetics along with monitored anesthesia care (MAC) (Figure 2).^12,22–28^

## MAC

Many cataract surgeries are performed under MAC, defined by The American Society of Anesthesiologists as “a specific anesthesia service performed by a qualified anesthesia provider for a diagnostic or therapeutic procedure. Indications include, but are not limited to, the nature of the procedure, the patient's clinical condition, or the need for deeper levels of analgesia and sedation than can be provided by moderate sedation (including potential conversion to a general or regional anesthetic).”^29^

Unlike general anesthesia, MAC does not require intubation or ventilation and can be titrated according to patient and surgeon needs.^30^ Approximately one-third of outpatient procedures in the United States, whether diagnostic or therapeutic, use MAC.^31,32^ The most common medications that comprise MAC in the United States are shown in Table 1.

**Table 1.** Medications That May Comprise MAC in the United States.

Drug class	Medication	Properties of note
Benzodiazepines	Midazolam	Short-acting; anxiolytic, amnesic, and sedative effects; no analgesic effects^22,33^
	Remimazolam	FDA approved in 2020; slow IV bolus^33^
	Diazepam	Potentially used for its anxiolytic and hypnotic effects; rapid onset of action (up to 30 min), but long half-life (20 to 80 h)^22^
IV anesthetic induction agents (nonbarbiturate sedative/hypnotics)	Propofol	Rapid onset, short-acting sedative/hypnotic; modest amnestic and antiemetic properties at subhypnotic doses; no analgesic effects^22,23,33,34^; associated with faster emergence from anesthesia^22^
	Etomidate	Rapid onset of action and short duration of sedation; fewer hemodynamic and respiratory effects; no analgesic effects^34^
	Ketamine (NMDA antagonist)	Produces a dissociative state with amnesia and intense analgesia; minimal respiratory depression at sedative doses (ketamine 0.25 to 0.5 mg/kg IV); often administered in small doses during MAC to enhance analgesia and decrease the need for opioids; rapid onset of action (∼1 min) and relatively short duration of action (10 to 20 min, with an elimination half-life of 2 to 3 h)^33^; downsides include cardiovascular stimulation, increased intraocular pressure, muscle hyperactivity, and emergence phenomenon^22^
Barbiturate	Methohexital	Rapid onset, ultrashort-acting barbiturate derivative used in IV doses for sedation^26,35^
Narcotic analgesics	Fentanyl (synthetic opioid)	Highly lipid-soluble opioid commonly used for sedation/analgesia^34^; small, intermittent IV boluses of 25 to 50 μg IV (reduced doses in older adults or when administered in combination with other sedatives/hypnotics); rapid onset of action (∼1-3 min) with a peak effect in approximately 6 min^33,34,36^; duration of action ∼30 to 60 min^33,36^ and half-life of 2-7 h^22^; no antianxiety and antiamnesia effects; quick passage across the blood–brain barrier^34^; emetic and depressive effects may be present postoperatively^37^
	Remifentanil	Only available as a powder that must be diluted for use; ultrashort acting with a very rapid onset of effect (60 to 90 s) due to the ability to cross the blood–brain barrier^22,33^; can be used to provide intense, titratable analgesia during stimulating portions of a procedure of any duration, without residual respiratory depression when stimulation is over^33^
Alpha-2-adrenergic agonist	Dexmedetomidine	Selective alpha-2-adrenergic agonist with sedative, anxiolytic, and modest analgesic effects^22,26,33,36^; for sedation, dexmedetomidine thought to have less respiratory depression than other sedatives^33^; administered intramuscularly or IV^22,26^; may decrease the need for propofol and/or opioids^36^

FDA = U.S. Food and Drug Administration; MAC = monitored anesthesia care

## OPIOIDS IN MAC

In the United States, approximately 99% of patients who undergo any surgery receive opioid analgesics at some point perioperatively.^38^ Fentanyl and remifentanil are the most commonly used opioids in MAC, and this decision is typically based on the clinicians' preference, duration of the procedure, cost, and convenience.

Opioids such as fentanyl remain a cornerstone of MAC during cataract surgery. A Mayo Clinic (Rochester, Minnesota) study analyzed sedation and recovery in 20 116 ophthalmologic procedures. Of these, 76.1% were cataract surgeries, with MAC being the most common (84.8%) anesthesia technique. Overall, 79.5% of ophthalmic surgery patients received fentanyl as part of their anesthesia protocol. Prolonged anesthesia recovery was linked to intraoperative fentanyl use, postoperative pain, and postoperative requirements for opioids. Of those patients with prolonged recovery, 97.2% had received fentanyl compared with 77.5% of patients who had an expected course of recovery. This study also found that perioperative opioids resulted in a prolonged duration of anesthesia.^39^ Part of the reason for this prolonged recovery with fentanyl is the increased incidence of postoperative nausea and vomiting associated with the use of opioids.^40^

Further evidence of the common use of fentanyl during cataract surgery can be seen by reviewing the clinical research literature, looking specifically at studies in which all cataract surgery patients receive fentanyl and those that use fentanyl as a comparator. The widespread use of both of these approaches in clinical trials speaks indirectly to the pervasive use of fentanyl in cataract surgery overall. A cursory search of PubMed for “cataract surgery” AND “fentanyl” yields results from both the ophthalmology and the anesthesiology literature. A representation of the publications of the last 20 years is summarized in Table 2.

**Table 2.** Sample of Clinical Trials From a PubMed Search Using “Cataract Surgery” and “Fentanyl”.

Year	Brief study description	Journal	Lead author
2003	Patients received retrobulbar anesthesia either with or without fentanyl.	*Journal of Cataract and Refractive Surgery*	Inan^41^
2004	Comparison between fentanyl in balanced salt solution and balanced salt solution with no analgesic.	*Journal of Clinical Anesthesia*	Aydin^42^
2008	Patients received midazolam with or without fentanyl.	*Acta Anesthesiologica Belgica*	Cok^43^
2008	All patients received midazolam and were randomized to receive either fentanyl or remifentanil.	*Saudi Medical Journal*	Cok^44^
2009	Comparison in pediatric patients of sub-Tenon block vs fentanyl.	*Anesthesia and Analgesia*	Ghai^45^
2012	All patients received fentanyl in combination with intraocular irrigation with balanced salt solution + epinephrine or balanced salt solution alone.	*Nepal Journal of Ophthalmology*	Miratashi^46^
2012	Comparison between fentanyl (control) and buprenorphine	*Saudi Journal of Anesthesiology*	Anaraki^47^
2015	All patients received midazolam and were randomized to receive fentanyl or acetaminophen.	*Archives of Anesthesiology and Critical Care*	Alipour^48^
2015	Comparison between propranolol and fentanyl + ketamine.	*Global Journal of Health Sciences*	Fazel^49^
2018	All patients received fentanyl and were randomized to also receive melatonin, acetaminophen, or placebo.	*Anesthesia and Pain Medicine*	Haddadi^50^
2019	All patients received fentanyl in combination with 1 of the following: etomidate, propofol, or midazolam.	*Anesthesiology and Pain Medicine*	Adinehmehr^34^
2020	Comparison between pethidine (meperidine) and fentanyl. All patients received propofol.	*Journal of Research in Medical Sciences*	Shetabi^51^

To assess whether opioids are used as part of routine institutional practice or MAC protocols in cataract surgery, a group from Duke University performed a single-center retrospective study of 2659 patients (3764 cases) undergoing routine cataract surgery over a 2-year period. Patients were excluded if they required combined surgery with a glaucoma, corneal, or retina procedure. The anesthesia for each case was reviewed, and it was found that 3649 cases (96.9%) received at least 1 dose of fentanyl.^51^ Although cataract surgery is often considered a minor, relatively low pain procedure, opioids are widely administered to patients undergoing cataract surgery.

## OPIOID CRISIS

Opioid pain medications were used for pain management in patients with cancer starting in the 1980s, when it was believed that the risk for addiction was rare.^52^ In the 1990s, pain experts pushed for pain to be considered the fifth vital sign.^53^ Between then and 2010, there was a 4 fold increase in opioid pain medication use and corresponding increases in overdose death rates and hospital admissions.^54^ From 1999 to 2016, over 200 000 deaths in the United States were attributed to overdoses involving prescription opioids.^55^

Most people addicted to opioids are first exposed through prescription medications.^56^ Both longer duration of treatment and higher dosages of opioids can increase the risk for OUD, overdose, and death. Although the annual prescribing rate decreased by 19% from 2006 to 2017, the amount of opioid in morphine milligram equivalents—a calculation of the total amount of opioids allowing for differences in drug type and strength—prescribed per person is about 3 times higher than in 1999. Looking at it a different way, approximately 58 opioid prescriptions were written for every 100 Americans in 2017, with an average of 18 days per prescription.^57^

A meta-analysis of 44 studies found that 61% of opioids prescribed after surgery are not used, resulting in large quantities of opioids potentially available in the community outside the context of surgery.^58^ In general, patients undergoing abdominal/pelvic and orthopedic procedures have fewer opioids left over than patients having nonabdominal soft tissue surgeries. In addition, open surgical procedures resulted in fewer leftover opioids than laparoscopic surgeries. The study concluded that postsurgical prescribing regulations for opioids should not be undertaken without considering the type and nature of the surgery.^58^ Although cataract surgery was not explicitly included in this analysis, one could surmise that phacoemulsification is a relatively low pain procedure, and opioids, if prescribed postsurgically, may go unused, thereby increasing the risk for diversion into the community.

## RISK FACTORS FOR OPIOID USE IN THE ELDERLY

The elderly cataract surgery patient population is not immune to this national crisis. Not only does opioid use pose a public health burden, but these elderly patients are also at an increased risk for OUD and for intraoperative and postoperative cataract surgery complications.^59^

There are several intertwining risk factors for opioid abuse. Age is a factor, with studies citing patients from age 50 to 80 years as being at risk for excessive opioid use.^1,3,60,61^ The mean age for cataract extraction in the United States is about 68 years.^62^ It has been shown that opioid misuse/abuse is growing fastest in the 50–64 year age group.^2,3^ For chronic pain or medical/surgical procedures, older adults are often prescribed opioids, which can lead to OUD. In addition, the elderly may experience anxiety, a decrease in independence, or the loss of a partner, any of which could potentially lead to a dependence on these medications.^3^ Older age may also result in accidental misuse due to forgetfulness, confusion, or impaired judgment due to polypharmacy.^2^

With over 300 million surgeries globally occurring each year, surgery is a risk factor for OUD, with postoperative pain a leading cause.^1,60,63,64^ Sun et al. analyzed inpatient and outpatient data from a large administrative health claims database. They assessed 11 surgical procedures between January 2001 and December 2013, identifying specific diagnosis and procedure codes as well as pharmacy claims. They included 641 941 opioid-naive surgical patients and compared them with 18 011 137 opioid-naive nonsurgical patients, looking at the incidence of chronic opioid use, defined as having filled ≥10 prescriptions or more than 120 days' supply of an opioid in the first year after surgery.^60^ Patients undergoing open cholecystectomy or total knee arthroplasty had the highest rates of chronic opioid use, with 1.2% and 1.4% incidence, respectively. Nonsurgical patients had a chronic opioid use rate of 0.136%. The procedures with the lowest rate of chronic opioid use after surgery were cesarean delivery (∼0.12%), cataract extraction (∼0.14%), and laparoscopic cholecystectomy (∼0.18%).^60^ Although the rate of chronic opioid use after cataract surgery was low, because this is the most common surgery worldwide, even a small percentage of potential patients with chronic opioid use result in a large number of patients at risk for OUD.^65–67^

Specific comorbidities may also increase the risk for OUD after surgery.^1^ A study analyzed 39 140 opioid-naive patients aged 66 years or older who underwent major elective surgery. It found that patients with diabetes, heart failure, and pulmonary disease had greater risk for prolonged opioid use.^64^ Because the cataract surgery population is older, they are more likely to have 1 or more of these comorbidities.

Another risk factor for OUD is simply age-related physiologic changes. The pharmacokinetics and pharmacodynamics of drugs can be vastly different in the elderly compared with younger patients.^2,40,66^ Geriatric patients tend to require smaller doses, have delayed onset of action, and experience prolonged effects of a drug. In addition, both cardiac and respiratory depression are more significant in this patient population.^40^ Overall, elderly patients are more sensitive to the adverse effects of opioids.^68^ It has also been found that the required dose of fentanyl decreased significantly with age, with 1 study noting a 50% decrease from age 20 to age 89 years and another estimating that opioid dosing should decrease by 25% to 50% in elderly patients.^42,68^ Paradoxically, elderly patients are often preferentially given fentanyl because of the risks from benzodiazepines such as respiratory depression, deeper sedation, delirium, and exacerbated dementia.^69^

## INCREASED RISK WITH INCREASED EXPOSURE

Several studies have shown that there is increased risk for addiction and overdose with each episode of opioid use.^70,71^ Elderly patients are more likely to have chronic conditions and temporally adjacent procedures, resulting in greater cumulative opioid exposure potential.^71^,^72^ With the cataract surgery patient population generally being over age 65 years, long-term opioid use and OUD are risks. Given that OUD risk increases with repeated exposures or cumulative doses, preventing any individual point of opioid exposure may reduce the overall medical risk.

## PAIN IN CATARACT SURGERY

It is argued that cataract surgery pain is present but poorly understood and potentially underestimated.^65,^^73–75^ Pain during cataract surgery is managed with MAC and other intraoperative pain management strategies; however, some patients still report moderate to severe discomfort. A 2015 study found that 35% of 106 patients undergoing first-eye cataract surgery reported intraoperative pain. This number was even higher in patients' second eyes, with 87% reporting pain during surgery.^76^ This may be due to increased awareness of the procedure leading to greater anxiety; thus, patients report greater pain.^77^

Another study assessed 63 patients undergoing phacoemulsification with just topical anesthesia and had patients grade their pain at different stages during surgery.^75^ Patients used the Keele verbal pain chart, grading their pain from 0 to 4 (none, mild, moderate, severe, and unbearable), toward the beginning, middle, and end of their cataract extraction. Analyzing scores from all surgical stages, 10.5% of patients experienced no pain throughout the procedure. Of those experiencing pain, the mean total pain score from all 3 stages combined was 3.05 (SD = 1.242) of 12. There were no statistically significant differences based on age, sex, or laterality. Reports of pain varied based on the cataract type, with patients having either corticonuclear + posterior subcapsular cataracts or white mature cataracts experiencing greater pain levels than those having posterior subcapsular cataracts alone.^75^

Postoperative pain has been assessed as well. Although most patients report no, slight, or mild discomfort after cataract extraction, there may be approximately 5% of patients who experience moderate to severe pain.^65,^^78^ A study in Finland analyzed 201 cataract surgeries in which only topical anesthesia was used. Postoperative pain was assessed, and 10% of patients reported ocular pain with a median pain score of 4 to 5/10 at 24 hours after surgery. By 6 weeks after surgery, 7% of patients reported ocular pain with a median pain score of 3 to 4/10.^78^

In another study, Porela-Tiihonen et al. systematically reviewed the incidence, prevalence, and management of pain after cataract surgery. Twenty-one studies were ultimately assessed, and some trends emerged. Whether assessed immediately postoperatively, within the first 72 hours, or weeks after surgery, pain was lower across the board in the groups receiving interventions to reduce inflammation and pain compared with the control groups. Having said that, the authors found that cataract surgery is associated with significant postoperative pain in some patients, with 1 study reporting the incidence of moderate or severe pain to be as high as 35%.^73^ This analysis concludes that although most recovery from cataract surgery is largely uneventful, a meaningful percentage of patients do experience significant postoperative pain that should be followed and managed.

With the widespread adoption of small incision phacoemulsification, the pain associated with cataract surgery has been minimized but not eliminated. Nonopioid pain relievers, such as nonsteroidal anti-inflammatory drugs (NSAIDs) and acetaminophen, can often control postoperative pain and discomfort in place of an opioid.^70^

## INTRAOPERATIVE OPIOIDS INFLUENCE POSTOPERATIVE OPIOID REQUIREMENTS

Opioids prescribed perioperatively may lead to long-term use, regardless of prior patient exposure to opioids.^79^ In some patients, more opioids used intraoperatively lead to a greater requirement for opioids postoperatively; this has been referred to as the opioid paradox.^38^ One may think that opioids intraoperatively would decrease the postoperative need for opioids, but this is not always the case. Although postoperative pain control is important, there is a known link between surgery and chronic opioid use; therefore, perioperative pain management decisions should be carefully considered.^64^

A study of U.S. adults of ages 18 to 64 years from a nationwide 2013 to 2014 insurance claims database assessed the incidence of new persistent opioid use after major or minor surgery.^80^ These patients were considered opioid naive, having filled no opioid prescriptions in the year before their respective procedures. For patients filling a perioperative opioid prescription, the incidence of persistent opioid use for >90 days was calculated, and the predictors of persistent opioid use after both major and minor surgical procedures were assessed. This study defined new persistent opioid use as an opioid prescription filled between 90 and 180 days after surgery. The 7109 patients undergoing major and 29 068 patients undergoing minor surgical procedures had a rate of new persistent opioid use from 5.9% to 6.5% compared with the nonsurgery control group, which had a rate of 0.4%.^80^ Although this study did not include cataract surgery, and the maximum patient age was 64 years, there could be some translation of these data to cataract surgery as a minor procedure.

Another study found that approximately 5% of patients were prescribed an opioid after cataract surgery.^63^ This study also assessed long-term risk for patients' dependence on opioids after low-risk surgical procedures. Patients undergoing cataract surgery who received a postoperative opioid prescription were approximately 60% more likely to be using opioids long term than those not receiving an opioid prescription. Of the low-risk surgeries assessed (cataract surgery, laparoscopy cholecystectomy, transurethral prostate resection, and varicose vein stripping), cataract surgery actually had the largest odds ratio for risk for long-term opioid use. Cataract surgery patients were 1.62 times more likely to use opioids long term compared with the other procedures, which ranged from 1.33 to 1.41 times.^63^

In a memorandum included in public comments to the Centers for Medicare and Medicaid Services' Calendar Year 2020 proposed rule for the hospital outpatient prospective payment system and the Medicare ambulatory surgical center payment system, David J. Clark, MD, PhD, Veterans Affairs Palo Alto and Stanford University Department of Anesthesiology, Perioperative Medicine, and Pain, stated, “Reducing exposure to opioids even after small surgeries may have even greater benefits in that it could reduce the cumulative risk resulting from multiple opioid exposures. Those in the elderly patient population may undergo multiple temporally related procedures (eg, cardiovascular procedures, total joint replacements, etc.), almost all of which entail opioid administration both during surgery and postoperatively.”^81^

Whether patients are taking opioids prior to surgery, simply undergoing surgery is a risk factor for chronic opioid use postoperatively. Assessing the surgical population as a whole, including patients taking opioids prior to surgery, postoperative chronic opioid use ranges from 9.2% to 13%.^76,79^ By decreasing the overall prevalence of chronic opioid use postoperatively, the goal is also to decrease opioid-related adverse events, which are the hallmark of the current opioid crisis.^79^

## OUTPATIENT OPIOID PRESCRIBING PATTERNS AMONG OPHTHALMOLOGISTS

Ophthalmology as a whole prescribes opioids at a lower rate (4%) compared with the national average across all prescribers (6.8%). Contrast these values to the median rates for surgeons as a whole (36.5%), pain medicine (48.6%), and physical medicine and rehabilitation (35.5%).^67^

A study analyzing the Medicare Part D Prescriber Data from 2013 to 2015 looked at opioid prescribing patterns among ophthalmologists. The study revealed that 88% to 89% of ophthalmologists wrote <10 opioid prescriptions annually. About 1% of ophthalmologists wrote 100+ opioid prescriptions per year. The average ophthalmologist wrote 7 opioid prescriptions per year with a 5-day mean supply.^70^ This study concluded that ophthalmologists are generally judicious in their prescribing patterns for opioids, but, given the opioid crisis, prescribing protocols should be reviewed as, even with a limited 5-day supply, up to 10% of patients can become chronic opioid users at 1 year.^70,^^82^

In another study, Kolomeyer et al. analyzed a large administrative medical claims database, identifying 2 407 962 incisional ocular surgeries from 2000 to 2016, which met strict enrollment criteria surrounding length of data available and timing of any opioid prescriptions. Of these patients, 45 776 were prescribed a postoperative opioid. The study assessed the rates of filled opioid prescriptions and found that the rate increased for all types of incisional ocular surgery over time.^71^ With the ongoing national opioid crisis, understanding these patterns may help ophthalmologists participate in addressing the epidemic.

Among ophthalmology subspecialties, cataract surgery had the highest number of filled opioid prescriptions in this limited database, with approximately 20 000 written during this 17-year period.^71^ Of these patients, 77% also received topical NSAIDs and/or steroids preoperatively, postoperative, or both. This suggests that topical treatments may not adequately address postoperative pain and highlights the need for alignment and coordination of cataract surgery pain management between ophthalmologists and anesthesiologists or other MAC providers.^72^

## DIVERSION OF OPIOIDS IN THE COMMUNITY

According to the Center for Behavioral Health Statistics and Quality, 50.5% of the estimated 4.3 million nonmedical users of prescription opioid drugs in 2014 obtained them for free from a prescription intended for a friend or relative, whereas 22.1% obtained them from a physician.^2,67,^^83^ This widespread community availability of opioids ultimately stems from the large supply of opioid prescriptions, thus contributing to the public health crisis. The quantity of opioids sold to hospitals, doctors' offices, and pharmacies increased by a factor of 4 from 1999 to 2010.^2^ In 2012, U.S. physicians wrote nearly 260 million opioid prescriptions, a staggering 70% of the global supply of opioids.^2,^^84^ Therefore, it is reasonable to expect that opioids given to patients may be opioids given to the entire community.

## NONOPIOID PAIN MANAGEMENT STRATEGIES IN CATARACT SURGERY

**Figure 3.**
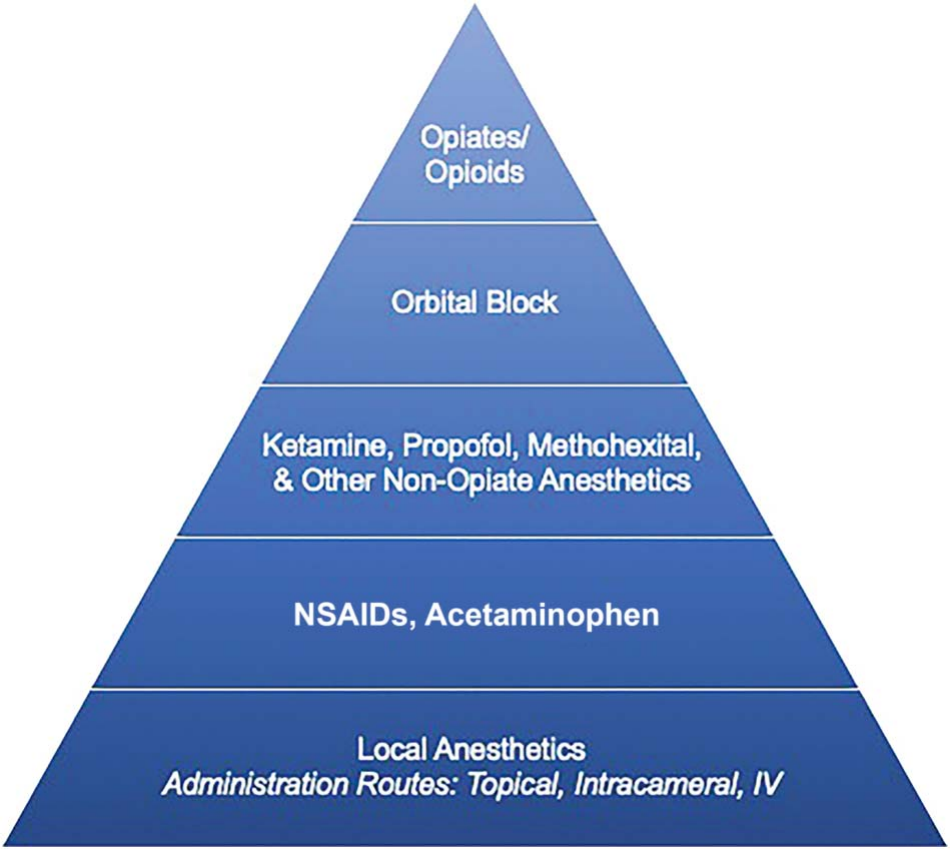
Pyramid of cataract surgery pain management. NSAID = nonsteroidal anti-inflammatory drug

Pain management during cataract surgery may be approached in a stepwise fashion. Figure 3 shows an algorithm in which opioids and opiates, although potentially necessary in some cases, can be used more sparingly as a later intervention. At the other end of the spectrum, cataract surgery may actually be performed without IV anesthesia. A study assessed 100 randomly selected patients from a 2-year period who received only topical anesthesia during cataract surgery.^15^ Patients received 1 drop of preservative-free 0.5% tetracaine, with additional drops or a tetracaine-soaked pledget if discomfort escalated. A change in patients' vital signs was used as a surrogate for pain. Patients' blood pressure increased by 1%, whereas the heart rate and respiratory rate decreased by 2% and 1%, respectively.^15^ Fichman concluded that overall, patients were very comfortable during cataract surgery despite receiving no IV sedation.

With the refinement of cataract surgery, some surgeons use this local/regional approach to pain without sedation.^36^ Providing patient education about the procedure and perioperative experience and setting realistic expectations may be adequate in addressing patient anxiety, which can decrease pain during surgery and lead to a decreased need for opioid pain management.^1,36^

Although eliminating sedation during cataract surgery may not work for all patients, there are other approaches that do not include opioids. Multimodal analgesia can be an effective pain management technique for cataract extraction in which 2 or more medications or nonpharmacologic interventions (eg, transcutaneous electrical nerve stimulation) having different mechanisms of action are used to address postoperative pain.^79,84^ Drugs or classes of drugs such as acetaminophen, NSAIDs (especially cyclooxygenase [COX]-2 inhibitors), gabapentinoids, corticosteroids, ketamine, and local or regional anesthetics can be used perioperatively and/or postoperatively to avoid some of the side effects of opioids, especially in the elderly cataract surgery population.^66,^^79^ The combination of treatments likely has an additive effect on opioid-sparing.^79,84^ Care should be taken, however, to consider the potential side effects of these alternative medications.

One option found to be an effective nonopioid sedative for cataract surgery is the sublingual MKO Melt (compounded by ImprimisRx), which contains midazolam, ketamine, and ondansetron. Jeffries et al. prospectively studied 611 patients undergoing cataract surgery by 2 surgeons. Patients were randomized to receive diazepam alone, diazepam/tramadol/ondansetron, or compounded midazolam/ketamine/ondansetron.^85^ All patients received topical anesthetic with additional anesthetic administered intracamerally (surgeon 1) or topically (surgeon 2). Although the midazolam/ketamine/ondansetron group showed a statistically significant reduced need for IV medications (*P* = .013) compared with the other groups, patients across all 3 groups required additional IV medications 32.1% of the time. No significant differences were found between surgical or discharge times across patient groups, surgeon or patient satisfaction, or side-effect profile between groups.^85^ The authors concluded that, “when used for cataract surgery, midazolam/ketamine/ondansetron offers an opioid-sparing alternative to conscious sedation that is safe, effective, and superior to diazepam in reduction of anxiety and need for IV medications.” It is important to note that compounded midazolam/ketamine/ondansetron has not been assessed for safety or efficacy by the U.S. Food and Drug Administration (FDA) and carries the patient risks inherent in compounded products.

An FDA-approved local nonopioid option specific to cataract surgery is Omidria [(phenylephrine and ketorolac injection) 1%/0.3% (Omeros Corporation)], a combination drug containing 1% phenylephrine, a mydriatic agent, and 0.3% ketorolac, an NSAID.^86^ Added to the irrigating solution for continuous intracameral administration during cataract surgery, phenylephrine/ketorolac 1.0%/0.3% minimizes intraoperative and postoperative (ie, total perioperative) opioid exposure while, at the same time, decreasing postoperative pain.

In 2 phase 3 prospective, randomized, double-blind clinical trials, 808 patients were randomized to receive treatment (n = 403) or placebo (n = 405) administered intracamerally with irrigation solution during cataract surgery. Mydriasis was maintained better in patients receiving phenylephrine/ketorolac 1.0%/0.3%, with the treatment group having a mean change in area under the curve of 0.08 mm compared with the placebo group, which had a change of −0.50 mm (95% CI, 0.51-0.65; *P* < .0001).^87^ There was also a significant reduction in early postoperative ocular pain measured via visual analog scale (VAS) scores. Within the first 12 hours, the VAS scores in the phenylephrine/ketorolac 1.0%/0.3% group were >50% lower than in the placebo group. In addition, the use of oral analgesics on the day of surgery was significantly lower in the treatment group compared with the placebo group (*P* = .001). The proportion of patients who were pain free at all measured time points was significantly higher, and concurrently, the proportion of patients experiencing moderate to severe pain at any time was significantly lower in the phenylephrine/ketorolac 1.0%/0.3% group.^87^

The ketorolac component of phenylephrine/ketorolac 1.0%/0.3% is an NSAID that inhibits both COX-1 and COX-2, which in turn inhibits surgical trauma-induced prostaglandin production downstream, thereby reducing postoperative inflammation and pain.^88^ Ketorolac has been demonstrated in therapeutic concentrations in the aqueous and vitreous up to 10 hours postdose in canines receiving intracameral phenylephrine/ketorolac 1.0%/0.3% compared with topical dosing.^89^ Consistent findings were noted after topical dosing in humans. Ketorolac is nearly undetectable in the aqueous immediately after cataract surgery and in the vitreous immediately after vitrectomy when preoperative ketorolac is administered topically.^90,91^ In contrast, intracameral phenylephrine/ketorolac 1.0%/0.3%, by remaining in the aqueous and vitreous well after the procedure is completed, contributes to the management of postoperative pain.

Similar pain management benefits of phenylephrine/ketorolac 1.0%/0.3% have been demonstrated in children. In a randomized, double-masked, active-control phase 3 pediatric study, mean pain scores, as measured by the Alder Hey Triage Pain Scale, were significantly lower in the phenylephrine/ketorolac 1.0%/0.3% group compared with the phenylephrine 1.0% group. Although this study was not powered to detect a difference in pain scores, significantly lower mean scores were seen at both 6 hours and 24 hours postoperatively (*P* = .029 and .021, respectively).^92^

Knowing that phenylephrine/ketorolac 1.0%/0.3% lessens patient pain, of interest is the impact it may have on decreasing perioperative opioid administration, particularly fentanyl, which is often used as part of MAC. Donnenfeld et al. compared intracameral phenylephrine/ketorolac 1.0%/0.3% to epinephrine (1 mg/mL) on pain reduction and opioid usage during cataract surgery. Sixty patients were prospectively enrolled, 41 in the phenylephrine/ketorolac 1.0%/0.3% group and 19 in the epinephrine group, all of whom received topical lidocaine gel, intracameral preservative-free lidocaine 1%, and a standardized regimen of preoperative and postoperative topical medications.^72^ If patients complained of pain intraoperatively, IV fentanyl was administered. Endpoints included pain as measured by the mean VAS score from 0 to 10, assessed 10 minutes postoperatively; administration of fentanyl intraoperatively; and a composite endpoint of patients considered responders who did not require fentanyl and had no to minimal pain (defined as ≤3 on VAS).

The mean VAS pain scores were 48.9% lower in the phenylephrine/ketorolac 1.0%/0.3% study group compared with the control epinephrine group (2.3 vs 4.5; *P* < .001). The proportion of patients with a VAS score ≤3 was greater in the phenylephrine/ketorolac 1.0%/0.3% group (85.0%) than that of the control group (31.6%; *P* < .001). Also, a significantly smaller proportion of patients in the study group (9.8% vs 42.1%; *P* = .006) required intraoperative fentanyl. With respect to the composite endpoint, the patients receiving phenylephrine/ketorolac 1.0%/0.3% were 94% less likely to require fentanyl or to have moderate to severe pain than those in the epinephrine group.^72^

Overall, intracameral phenylephrine/ketorolac 1.0%/0.3% led to an almost 80% decrease in the need for opioids intraoperatively and, concurrently, an approximate 50% decrease in mean VAS pain scores. The authors of this study postulated that the patients in the study group experienced less pain as a result of the ketorolac but may have also experienced less pain because of the phenylephrine providing dilation sufficient to decrease the need for iris manipulation, thereby additionally decreasing the resultant pain. Phenylephrine/ketorolac 1.0%/0.3% also allowed for a 13% reduction in surgical time, further decreasing the potential for tissue manipulation, prostaglandin release, and patient pain.^72^

In a subsequent prospective, single-center, randomized, double-masked, self-controlled clinical trial, Donnenfeld again compared pain control and the need for fentanyl during cataract surgery with intracameral use of either phenylephrine/ketorolac 1.0%/0.3% or epinephrine during cataract surgery. In this study, a total of 112 eyes of 56 patients undergoing bilateral cataract surgery were enrolled, with 1 eye randomized to treatment and the other to epinephrine as the control.^93^

Overall, the number of patients requiring intraoperative fentanyl analgesia was significantly lower in the phenylephrine/ketorolac 1.0%/0.3% group compared with the epinephrine group (12.5% vs 33.9%; *P* = .013). Mean VAS pain scores intraoperatively were also significantly (56.7%) lower in the phenylephrine/ketorolac 1.0%/0.3% group than in the epinephrine group (1.3 vs 3; *P* < .001), as were the mean VAS pain scores at 10 minutes (0.9 vs 2.1; *P* < .001) and 1 day (0.2 vs 0.6; *P* < .001) postoperatively, representing pain score reductions of 57.1% and 66.7%, respectively.^93^

## MINIMIZING PRESURGICAL AND POSTSURGICAL OPIOIDS

In addition to perioperative opioid considerations, prescribing patterns for postsurgical opioids is important, too. Jackson et al. looked at real-world opioid prescribing after cataract surgery in patients who received phenylephrine/ketorolac 1.0%/0.3%. This retrospective study assessed data from the MarketScan databases of patients undergoing cataract surgery with or without phenylephrine/ketorolac 1.0%/0.3%. A group of 218 672 patients was identified from the data between January 1, 2015, and July 15, 2019. Of these, 5145 received phenylephrine/ketorolac 1.0%/0.3% during surgery. In the 2-day postoperative period, 0.50% of the phenylephrine/ketorolac 1.0%/0.3% group and 0.68% of the 213 527 control group patients (*P* = .135) filled at least 1 opioid prescription. The opioid pill count for the first prescription for patients receiving phenylephrine/ketorolac 1.0%/0.3% was significantly lower than for patients in the control group, with 20 pills and 45 pills prescribed, respectively (*P* = .015). The findings were similar when assessing a 7-day postoperative window. Despite demonstrating a statistically significant lower number of opioids prescribed, the phenylephrine/ketorolac 1.0%/0.3% patients, as a whole, were also significantly more likely to have 1 or more presurgical comorbidity or risk factors for complex surgery than the control group (46.6% vs 31.3%; *P* < .001), which would be expected to have resulted in increased postoperative pain.^94^

Overall, this study demonstrated a reduction in the quantity of opioids prescribed for patients receiving intracameral phenylephrine/ketorolac 1.0%/0.3% during cataract surgery despite these patients having greater burden of both comorbidities and surgical risk factors. The investigators hypothesize that the use of phenylephrine/ketorolac 1.0%/0.3% intraoperatively may decrease patient exposure to opioids after cataract surgery and the volume of pills that may be diverted into the community.^94^

Although perioperative opioid use can lead to intraoperative and postoperative issues, preoperative opioid use can cause problems during cataract surgery as well. A study from Kresge Eye Institute assessed consecutive cataract surgery patients from January 1, 2017, to April 30, 2018, noting patient self-reports about whether they were using a prescription opioid at the time of surgery. Rodriguez Torres analyzed intraoperative complications, both minor and severe, as well as visually significant or vision-threatening complications within a 90-day period postoperatively. A total of 169 patients were assessed, 26 of which were using an opioid at the time of surgery; the opioid user and nonuser groups were similar demographically.^59^

The overall rates of intraoperative complications (odds ratio 5.018, 95% CI, 1.250-20.140, *P* = .013) and postoperative complications (odds ratio 3.068, 95% CI, 0.851-11.058, *P* = .074) were higher in the opioid users than the nonusers. Both visually significant and vision-threatening complications were higher in the prescription opioid user group as well. Overall, prescription opioid users were 5 times more likely to experience intraoperative complications during cataract surgery and 3 times more likely to have postoperative complications.^59^

As the opioid crisis continues, a number of surgical specialties have looked at their opioid prescribing patterns. As discussed, there have been just a few studies looking at prescribing trends within ophthalmology, focusing primarily on prescriptions written without looking at how many are actually filled.^67,70^ The Kolomeyer study found that the overall rate of opioid prescriptions filled after cataract surgery was increasing from 2000 to 2016. During that period, opioid prescriptions were filled following 0.95% per cataract surgery procedure.^71^ Ocular incisional surgeries as a group were associated with a 1.9% rate of filled opioid prescriptions; however, the greatest number of surgeries performed in this group were cataract extractions, which, when excluded, resulted in a 7.57% rate of filled opioid prescriptions across the remaining ocular incisional procedures, collectively.^71^

Of interest, although cataract surgery techniques, equipment, and technology have advanced, resulting in less invasive procedures, both patient-perceived pain and opioid prescriptions are increasing. Kolomeyer et al. postulated several potential reasons for this paradox, including deficient physician education about opioid prescribing or pain assessment, greater focus on pain as the fifth vital sign, lack of state and/or national standardization of opioid prescribing regulations, increased awareness by physicians about patient pain/pain perception and the correlation with physician and hospital satisfaction scores, and drug company marketing strategies.^71^

The authors of the Kolomeyer study encouraged ophthalmologists to evaluate their opioid prescribing habits. Particularly in light of the national opioid crisis, it is time for ophthalmologists as a whole, including cataract surgeons, to assess the role of opioids in pain management for cataract surgery. With nonopioid pain management strategies such as multimodal anesthesia, phenylephrine/ketorolac 1.0%/0.3%, postoperative topical pain medications, and even patient education, cataract surgeons can positively influence patients away from opioids.

## PUBLIC HEALTH AND SYSTEMIC STRATEGIES

The U.S. government's public health response to the opioid crisis has focused on stopping abuse, decreasing the opioids diverted into the community, and minimizing demand.^67^ One example of a systemic strategy is the Veterans Administration's approach to addressing OUD, developed in 2017, called S.T.O.P. P.A.I.N., a comprehensive, multipronged approach to prevent opioid abuse and treat those who become addicted.^3^

**S**–Stepped Care Model

**T**–Treatment alternatives/complementary care

**O**–Ongoing monitoring of usage

**P**–Practice Guidelines

**P**–Prescription monitoring

**A**–Academic Detailing

**I**–Informed consent for patients

**N**–Naloxone distribution

There have been several legislative approaches as well, such as the 2018 Substance Use-Disorder Prevention that Promotes Opioid Recovery and Treatment for Patients and Communities Act which, among other provisions, directed the Center for Medicare and Medicaid Services to review payment structures to remove financial incentives for practitioners to use and prescribe opioids instead of nonopioid alternatives.^95^ Another bill that generated strong bipartisan support in the 116th Congress in both the Senate and the House of Representatives and has been reintroduced in both chambers of the 117th Congress is the Non-Opioids Prevent Addiction In the Nation Act.^96,97^ If passed, the Non-Opioids Prevent Addiction In the Nation Act would amend the Social Security Act to combat the opioid crisis by promoting access to nonopioid treatments in the hospital outpatient setting. Each of these public health measures appropriately seeks to address an important aspect of the opioid epidemic.

## LIMITATIONS TO CURRENT KNOWLEDGE

Although much has been learned about the opioid crisis and how cataract surgeons can respond, there are some limitations and gaps in the current data. There have been no comprehensive studies assessing pain management and analgesia at all points in the cataract surgery process, nor have there been studies assessing the prescribing patterns by provider type and the ways in which ophthalmologists and anesthesiologists could more effectively collaborate, providing patients the best possible surgical experience and outcome without exposing them, or their communities, to the risks of opioid medications.

## THE ROLE OF CATARACT SURGEONS IN THE BATTLE AGAINST THE OPIOID CRISIS

Although cataract surgeons do not prescribe opioids for postoperative pain at a high rate, the sheer volume of cataract surgeries performed both in the United States and worldwide means that there are a large number of patients who receive and fill opioid prescriptions, with which comes the risk for potential opioid misuse, abuse, and diversion. There are several ways that cataract surgeons can actively combat the opioid crisis. First, understand that there is a persistent disconnection between pain management and patient pain experience in cataract surgery despite increasingly refined surgical techniques and take the opportunity to assess, change, and improve standard care practices. Second, take a holistic approach when caring for these elderly cataract surgery patients. By doing so, cataract surgeons can not only mitigate the risk for OUD but also help prevent polypharmacy issues in this population. Third, be particularly mindful of cataract surgery patients already on an opioid regimen for another condition and take mitigating steps to decrease the potential for complications. Finally, reevaluate your standard protocols for pain management and analgesia surrounding cataract surgery and consider using FDA-approved nonopioid alternatives such as phenylephrine/ketorolac 1.0%/0.3% and, if and when approved, midazolam/ketamine/ondansetron; nonpharmacologic support; or some combination of these nonopioid strategies. The goal of ophthalmologists should be to balance patients' pain control needs with judicious use of opioids while better using newer and safer nonopioid alternatives. This is an excellent opportunity to collaborate with anesthesia colleagues to educate each other about what joint practices might best serve cataract surgery patients.

## CONCLUSION

There are numerous potential issues arising from the use of perioperative opioids in the cataract surgery patient population. Not only these elderly individuals are more sensitive to the adverse effects of opioids but also their risk for OUD may be inherently increased by both number of exposures and total cumulative doses of opioids.^42,68,70,71^,^72^ Use of intraoperative opioids can increase postoperative opioid requirements, and cataract surgery patients receiving an opioid prescription are 60% more likely to use opioids long term than those who are not prescribed an opioid.^38,63^ Patients given a prescription for an opioid medication may leave all or part unused, thereby opening the potential for diversion of those pills into the community.^58,^^98^

By minimizing the need for an opioid, whether perioperatively or postoperatively, there will be broad benefits. It has been shown that phenylephrine/ketorolac 1.0%/0.3% results in the need for fewer opioids and less pain, and fewer opioids result in fewer complications, all collectively improving patient satisfaction.^72^,^82,87^ Fewer complications, shorter surgeries, and less time in the postanesthesia care unit have positive financial implications.^39^,^72^,^82^ Finally, there are public health implications associated with the use of phenylephrine/ketorolac 1.0%/0.3% in that decreased need for opioid prescriptions postoperatively means potentially less risk for OUD for cataract surgery patients and fewer opioids susceptible to diversion within our communities.^58^ Ophthalmology can make a difference to patients individually and to society as a whole by judicious use of opioids and using newer and safer nonopioid alternatives.
